# Adverse Reactions to Evolocumab: Analysis of Real-World Data from EudraVigilance

**DOI:** 10.3390/ph17030364

**Published:** 2024-03-11

**Authors:** Fabrizio Calapai, Carmen Mannucci, Mariaconcetta Currò, Luigi Cardia, Emanuela Esposito, Gioacchino Calapai, Ilaria Ammendolia

**Affiliations:** 1 Department of Clinical and Experimental Medicine, University of Messina, 98125 Messina, Italy; fabrizio.calapai@unime.it (F.C.); mariaconcetta.curro@unime.it (M.C.); ilaria.ammendolia@unime.it (I.A.); 2 Department of Biomedical and Dental Sciences and Morphological and Functional Imaging, University of Messina, 98125 Messina, Italy; cmannucci@unime.it; 3 Department of Human Pathology of Adult and Childhood “Gaetano Barresi”, University of Messina, 98125 Messina, Italy; luigi.cardia@unime.it; 4 Department of Chemical, Biological, Pharmaceutical and Environmental Sciences, University of Messina, 98125 Messina, Italy; emanuela.esposito@unime.it

**Keywords:** evolocumab, adverse reactions, pharmacovigilance, PCSK9 inhibitors, EudraVigilance

## Abstract

Background: Evolocumab is a humanized immunoglobulin G2 monoclonal antibody, directed against Proprotein Convertase Subtilisin/Kexin type 9 (PCSK9), prescribed in hypercholesterolemic patients. The safety profile of this drug is currently defined by the data of pre-authorization clinical trials. The purpose of this study is to update knowledge of the safety of evolocumab through an analysis of post-marketing real-world data on suspected adverse reactions (SARs), reported by the EudraVigilance database system. Methods: The public version of the EudraVigilance database has been used, and only serious SARs signals were included. Results: Musculoskeletal system disorders, flu-like symptoms, injection-site reactions, skin reactions, and metabolism and nutrition disorders are observed in the post-marketing surveillance, as well as being found in the pre-authorization studies. Not previously signaled in the pre-marketing studies, diarrhea was reported. Furthermore, signals related to cardiac adverse reactions, more frequently at the expense of adults in comparison to elders, were found. Conclusions: The post-marketing safety profile of evolocumab emerging from an analysis of the EudraVigilance data system indicates it is sufficiently safe but suggests the necessity for caution when it is prescribed to hyperlipidemic patients affected by heart diseases.

## 1. Introduction

Evolocumab is a selective humanized immunoglobulin G2 (IgG2) monoclonal antibody, directed against Proprotein Convertase Subtilisin/Kexin type 9 (PCSK9) and produced with recombinant DNA technology. Evolocumab selectively binds PCSK9, a secreted serine protease involved in cholesterol homeostasis, inducing low-density lipoprotein receptors (LDL-R) degradation, and according to this mechanism, it plays a key role in hypercholesterolemia, in platelet aggregation, and in the development of atherosclerotic plaque and aortic valve calcification (Qi), because it binds low-density lipoprotein receptor-related protein 1 (LRP1), apolipoprotein E receptor-2 (ApoER2), and very-low-density lipoprotein receptor (VLDL-R) [1]. Moreover, PCSK9 seems to be involved in the physiopathology underlying the development of pancreatic cancer and Alzheimer’s disease [2,3]. PCSK9 is an enzyme especially synthesized by hepatocytes, but also in the kidney, lung, spleen, thymus, small intestine and nervous tissue [4]. Successively, it enters the circulation and binds to the epidermal growth factor-like repeat A domain of the LDL receptor (LDL-R), thus reducing the liver’s capacity to bind and remove low-density lipoprotein cholesterol (LDL-C), and through the acceleration of LDL-receptor lysosomal degradation, it increases blood LDL-cholesterol levels [5,6]. Physiologically, LDL-R presents a domain for Apolipoprotein B-100 (Apo-B) to bind LDL-C and for the internalization of the LDL/LDLR complex. This process causes LDL-R return to the cell surface by a recirculation mechanism, while free C-LDL is transported to lysosomes, where it is degraded to lipids and amino acids [7].

Patients diagnosed with hypercholesterolemia and mixed dyslipidemia doubled in the decade 2009–2019, with a large number of these people being at a high or very high risk of cardiovascular disease [8]. The active principle evolocumab has shown significant efficacy in reducing C-LDL levels by up 75%; this result has been confirmed by a phase 3 trial which demonstrated that evolocumab treatment, in combination with diet and a low dose or high dose of atorvastatin, caused a reduction in LDL cholesterol levels, ApoB levels, and triglycerides levels in a 52-week placebo-controlled trial conducted by enrolling patients at cardiovascular risk [9]. In another study investigating the pharmacokinetics and pharmacodynamics of this substance, a reduction in C-LDL was detected after a single subcutaneous administration of evolocumab, with peak LDL-C reduction by 14 and 21 days [6].

This monoclonal antibody is prescribed for patients who do not control cholesterol levels with diet. It was licensed in Europe on 17 July 2015 for subcutaneous use with the following therapeutic indications: in patients affected by “Hypercholesterolemia and mixed dyslipidemia”, “Homozygous familial hypercholesterolemia”, and “Established atherosclerotic cardiovascular disease”. The safety profile of this monoclonal antibody is still currently defined by results obtained with clinical trials used for its authorization of entry in the market and highlighted in its summary of product characteristics (SPC) [10]. A randomized, controlled phase III clinical trial showed that evolocumab’s efficacy as monotherapy every 4 weeks over one year of treatment to decrease C-LDL is also associated with minor side effects [11]. The OSLER-1 trial, evaluating the longer-term effects of evolocumab during an open-label hypercholesterolemia treatment 5-year study, showed the LDL-C-lowering efficacy, tolerance, and safety of evolocumab, with no formation of neutralizing antibodies, throughout the period of the clinical research [12]. Other clinical studies report that this drug is well tolerated, with most common side effects including upper respiratory tract infections, nausea, and injection site reactions [13]. However, musculoskeletal and connective tissue disorders, particularly myalgia, seem to be very common, as shown by “real-world” data collected with randomized clinical trials [14]. However, it has been suggested that, based on a cost much higher than the existing medicinal products and the low incremental mortality benefit, evolocumab could be considered to have an unfavorable cost–benefit ratio [15]. According to the SPC published by the EMA, common side effects are the occurrence of flu (high temperature, sore throat, runny nose, cough, and chills), the common cold, such as a runny nose, sore throat, or sinus infections (nasopharyngitis or upper respiratory tract infections), nausea, back pain, joint and muscle pain, injection site reactions, allergic reactions including rash, and headache. Urticaria was signaled through clinical trials conducted before drug registration. In light of the above data, the purpose of this study is to confirm the pre-authorization profile or to highlight new aspects of the safety of the drug evolocumab in patients with homozygous familial hypercholesterolemia and dyslipidemia, through the analysis of post-marketing real-world data on suspected adverse reactions (SARs) to this drug, reported by the EudraVigilance database system in the years 2020–2021–2022–2023.

## 2. Results

A total of 1148 ICSRs and 209 reports of serious cases (serious cases = 18.2% of total ICSRs) related to evolocumab are traceable in EudraVigilance. The sex distribution of ICSRs shows that the reports sent to EudraVigilance are equally divided between females and males, and only one case of death was detected among the reports of serious cases (Table 1).

According to an analysis of the frequency of PTs, the more frequent serious adverse reactions suspected to be caused by evolocumab are, in descending order, myalgia, flu-like symptoms, diarrhea, joint pain, back pain, urticaria, and angioedema. The remaining SARs are listed in Table 2.

Since for each ICSR more than one SAR can be included, the number of SARs (258) is largest than number of ICSRs (*n* = 209). Analysis of the SARs using the System Organ Class (SOC) level of adverse reactions hierarchy shows that musculoskeletal and connective tissue disorders, general disorders and administration site conditions, skin and subcutaneous tissue disorders, gastrointestinal disorders, vascular disorders, and cardiac disorders are the most frequent reaction groups to be signaled. The complete list of reaction groups according to the SOC level is included in Table 3.

An analysis of the sex distribution of SARs at SOC level shows apparently different results between males and females. In particular, a percentage of 56.6% of SARs are females, but when statistics was applied, no difference between the sexes was observed for each SOC level. However, a calculation of the odds ratio for the risk of occurrence of a number of adverse reactions more than one for single cases indicated that females are at greater risk to experience this (Table 4).

Results analyzed according to the age distribution show that 18–64 and 65–85 years are the range of ages principally of interest in the occurrence of adverse reactions to evolocumab. In this context, there is no difference in the frequency of adverse reactions, except for the cardiac disorders SOC level, more frequent in adults (18–64 years) (Table 5).

In Table 6, the results of the disproportionality test, calculated as the odds ratio comparing serious SARs to evolocumab with the serious SARs of the other Proprotein Convertase Subtilisin/Kexin type 9 inhibitor, alirocumab, signaled in the years 2020–2021–2022–2023, are reported. The risk of reporting SARs to evolocumab is >1, compared to the same risk for alirocumab for vascular disorders, cardiac disorders, metabolism and nutrition disorders, psychiatric disorders, ear and labyrinth disoders, and blood and lymphatic disorders.

## 3. Discussion

The aim of this research is to contribute to the knowledge of the safety of evolocumab in patients treated for homozygous familial hypercholesterolemia and dyslipidemia. Information on the post-marketing period helps to update and to stabilize what we know about evolocumab and represents a contribution for a better use of this drug in clinical practice. The present descriptive analysis presents a partial confirmation of the pre-marketing studies and raises new less-known aspects raised through the view of post-marketing real-world data related to this drug, collected by EudraVigilance. The pre-marketing information on adverse reactions, used to obtain the authorization of evolocumab as a medicinal product together with data on efficacy, reported as common adverse reactions the following: influenza, nasopharyngitis, respiratory tract infections, and more hypersensitivity and rash, respectively, for the SOC levels infections and infestations and immune system disorders. Urticaria, identified at the SOC level Immune disorders, was considered an uncommon adverse reaction to evolocumab. Other common disorders included in the summary of the characteristics of the product released with the drug were headache and nausea, for nervous system disorders and gastrointestinal disorders, respectively. On the safety information released with the market authorization were also backpain, arthralgia, and myalgia, belonging the SOC level termed musculoskeletal and connective tissue disorders. Injection site reactions were considered common, while influenza-like symptoms, also included in the SOC level “General disorders and administration site conditions” were reported as uncommon reactions. The occurrence of angioedema, included in the SOC level “Skin and subcutaneous tissue disorders”, was classified as a rare adverse reaction among those caused by evolocumab (summary of product characteristics). A recent analysis of potential adverse reactions to this drug carried out through data mining of the spontaneous reporting system of the Food and Drug Administration, FDA Adverse Event Reporting System (FAERs), concludes that evolocumab is safe and exhibits a safety profile. The results of this study, through a disproportionality analysis, focused particularly on injection site reactions and muscle-related adverse events related to evolocumab prescription [16]. Another study on real-world data, using the same data system, reported similar conclusions about musculoskeletal problems and injection site reactions; however, the authors added influenza-like illness as a significant signal of adverse events to evolocumab [17]. In patients at extremely high risk for acute coronary syndrome, with elevated low-density lipoprotein cholesterol levels and subjected to percutaneous coronary intervention and treated with 140 mg evolocumab every two weeks, associated with atorvastatin 40 mg/day and ezetimibe 10 mg/day, lipids were decreased, with an improvement in cardiovascular prognosis, without any further adverse reaction. However, the limit for this study was the short duration of treatment (three months) [18].

Results from a disproportionality analysis of PCSK9 inhibitors using the FAERS database, and applying the information component and the reporting odds ratio about the difference in adverse reactions between evolocumab and alirocumab, identified a possible signal of ocular disorders associated with PCSK9 inhibitors without any difference [19]. The association between the use of PCSK9 inhibitors, with or without statins, and the risk of musculoskeletal adverse events was investigated through an analysis of ROR as an index of disproportionality based on FAERS data. Results showed that the use of PCSK9 inhibitors such as evolocumab is associated with musculoskeletal adverse events and that this risk is enhanced with an association with statins [20].

Together with the data observed in the present research, these results suggest the need for a comment on the possible mechanistic principles causing muscular disorders induced by PCSK9 inhibitors. Muscle-associated adverse events caused by PCSK9 inhibitors were reported only in a few clinical studies, including little samples of patients. Moreover, the prevalence of muscle-associated adverse events related to PCSK9 inhibitors is not known. Muscle pain due to antihyperlipidemic drugs is not fully understood, even though a statin-induced depletion of ubiquinone has been proposed as the principal mechanism. Unfortunateley, this hypothesis does not seem valid for muscle disorders induced by PCSK9 inhibitors or ezetimibe, since they do not deplete ubiquinone. Furthermore, it has been observed that muscle disorders induced by PCSK9 inhibitors are evident later in the treatment, with respect to statins [21].

The study FOURIER (Further Cardiovascular Outcomes Research with PCSK9 Inhibition in Subjects with Elevated Risk) is a randomized, double-blind study, controlled with a placebo, with 27,564 patients affected by atherosclerotic cardiovascular disease receiving a treatment with statins and evolocumab. During the period of the study (about 2.2 years), the occurrence of cardiovascular adverse reactions was evaluated. The results of this clinical research did not show significant differences in the cardiovascular event rate across the ages, but according to the authors’ conclusions, adverse reactions were more common in women and increasing with age [22].

Another observational, retrospective, descriptive, and multicenter study conducted for 24 months on 90 patients with hypercholesterolemia, receiving 140 mg evolocumab every two weeks, found that the frequency of cardiovascular adverse reactions was low. In particular, it detected a light significant increase in injection site reactions in the youngest age in men with respect to women [23].

Adverse reactions concerning the musculoskeletal system, flu-like symptoms, injection site reactions, and skin reactions are also confirmed to be more frequent among serious SARs (the category investigated in the present analysis). Instead, it could be considered unusual that diarrhea was found to be one of the most frequent serious adverse reactions, without being signaled in the summary of product characteristics of evolocumab or other clinical studies investigating the safety profile of this drug. Another issue of interest is the rate of cardiac disorders related to evolocumab observed in our analysis. Evolocumab has been approved by the FDA after several clinical trials (OSLER, FOURIER) that consistently showed favorable cardiovascular outcomes with a positive safety profile [24]. Evolocumab is known to lower the risk of cardiac disorders in people with atherosclerotic cardiovascular disease, and cardiac adverse reactions are not signaled with the evolocumab authorization, thus suggesting that these events became evident only after the post-marketing exposure of patients. However, it can be associated with rare side effects, and a case of atrial fibrillation as a possible adverse reaction has been reported by post-marketing surveillance in a 69-year-old woman [25]. EudraVigilance data indicate that serious cardiac disorders related to evolocumab are significantly more frequent in adults in comparison to elder patients. No significant difference between men and women in the rate of cardiovascular events was observed in the EudraVigilance data. However, the greater vulnerability of women exposed to adverse reactions potentially caused by evolocumab is here revealed by the larger number of single adverse reactions occurring in single cases.

The limitations of this study are typical of research based on spontaneous reporting databases of adverse drug reactions. They are represented principally by weaknesses due to the poor quality of single signals and the lack of a denominator. Furthermore, it is generally recognized that other limitations, such as under-reporting and Weber and notoriety effects, are an obstacle to producing definitive conclusions by using this method [26].

## 4. Material and Methods

EudraVigilance is a database containing SARs linked to drugs yet licensed for the market or being currently studied in clinical trials in the European Union (EU). In this data system, SARs are described in single Individual Cases Safety Reports (ICSRs), and they are signaled by healthcare professionals or non-healthcare professionals to their own EU national competent authorities or to marketing authorization holders. In this study, only ICSRs reporting SARs to evolocumab signaled by healthcare professionals from 1 January 2020, up to 31 December 2023 were analyzed. EudraVigilance collects signals reports to suspected adverse reactions, not desired medical events that have been observed following the use of a medicine, but which are not necessarily related to or caused by the same medicine [27].

For this article, the public version of the EudraVigilance database has been used and only reports from EEA countries and the United Kingdom (UK) were evaluated. EudraVigilance allows access to reports of suspected side effects/adverse drug reactions submitted electronically by national medicines regulatory authorities and pharmaceutical companies [28]. Information on patient characteristics (age group and sex), type of adverse reaction (there are often more than one for each ICSR), primary source qualification, and concomitant drugs were provided for all reports. The terms “sex” and “gender” are used interchangeably here because only the “sex” field is available in EudraVigilance and the information collected refers to biological sex.

Regarding selection criteria of data, ICSRs reporting SARs were selected on the basis of the Medical Dictionary for Regulatory Activities (MedDRA). MedDRA is a standardized, clinically validated international medical terminology used by regulatory authorities and the biopharmaceutical industry. It is used for coding cases of adverse effects in pharmacovigilance databases and to facilitate searches in adverse drug reactions databases (MedDRA) [29]. Adverse reactions were described by using “Preferred Terms” (PTs) listed in MedDRA. PT is a distinct descriptor (single medical concept) for an adverse symptom or an adverse sign. Two or more PTs having an overlapping clinical meaning were aggregated to avoid not-useful duplicate PTs with the same connotation (for example, the two PTs myalgia and muscle pain). MedDRA has a hierarchy of terms to describe adverse reactions. Adverse reactions are assembled into the SOC terms in the MedDRA hierarchy such as musculoskeletal and connective tissue disorders, vascular disorders, etc. The System Organ Class is the highest level of the hierarchy that denotes the largest concept useful to retrieve data [30].

Data were analyzed after the aggregation of PTs of single reports in a higher level of the MedDRA hierarchy by the merging of single serious SARs in the level of the SOC (for example, nausea and vomiting classified in the same group of gastrointestinal symptoms). In accordance with the International Council on Harmonization E2D guidelines, ICSRs are classified as serious if they are life-threatening, resulted in death, caused/prolonged hospitalization or disability, or related to a congenital anomaly/birth defect or other medically important condition. An appropriate stratification of signals by groups for age and sex was performed to avoid bias caused by confounding effects and to analyze in a distinct way these two variables. Duplicate and incomplete ICSRs were excluded from the analysis. A descriptive statistical analysis was performed in the present research. The odds ratio (OR) was used to evaluate the level of odds of reporting more adverse events for single cases between males and females. The Chi-square (χ^2^) test was applied to evaluate the association of adverse reactions SOC level and age or sex. *p* was considered significant when <0.05. The risk of false positives due to multiple testing for statistical significance was reduced by the application of a post hoc Bonferroni correction.

A disproportionality analysis was performed based on the reporting odds ratio (ROR). Here, the ROR is used to establish the strength of disproportionality. Reports of serious SARs to evolocumab were compared with serious SARs of the only other Proprotein Convertase Subtilisin/Kexin type 9 inhibitor, alirocumab [31], signaled in the years 2020–2021–2022–2023. A ROR equal to 1 states the absence of a signal; conversely, a ROR greater than 1 indicates a signal and the existence of an association. The higher the ROR, the stronger the association. The ROR is statistically significant when the lower bound of its 95% CI is greater than 1 [32,33]. The statistical software SPSS version 29.0.0.0 (190) was used for the analysis of data. Ethical approval was not needed because the research was performed using anonymized public data.

## 5. Conclusions

Despite the above cited limitations, the present analysis of the signals of SARs to evolocumab sent during the last four years to the EudraVigilance data system indicates a partial confirmation of the safety profile of the drug, as it is highlighted in documents related to its market authorization. In particular, musculoskeletal system disorders, flu-like symptoms, injection site reactions, skin reactions, and metabolism and nutrition disorders are observed in the post-marketing surveillance, as well as having happened during pre-authorization studies. There is confirmation of adverse episodes of nausea, but, in the context of gastrointestinal reactions, there appear several signals, not previously signaled in pre-marketing studies with evolocumab, reporting the symptom diarrhea. Moreover, it is noteworthy that there is a presence in the data system of signals related to cardiac adverse reactions, more frequently at the expense of adults in comparison to elders.

A disproportionality analysis comparing data on the risk of reporting serious suspected adverse reactions indicates an evolocumab risk profile slightly less safe than the other PCSK9 inhibitor, arilocumab. Finally, although there are no statistically significant differences in the frequency of the number of adverse reactions to evolocumab signaled for men and women, a form of susceptibility of women was detected, as evidenced by a greater number of symptoms or adverse reactions per single case appearing in women compared to men. In conclusion, the post-marketing profile of evolocumab emerging from the analysis of SARs contained in the EudraVigilance data system indicates that this drug is sufficiently safe but suggests the necessity of further caution when it is prescribed to hyperlipidemic patients affected by heart diseases and the management of adverse reactions occurring in women.

## Figures and Tables

**Table 1:** Serious suspected adverse reactions (SARs) related to the prescription of evolocumab in European Economic Area (EEA) and United Kingdom according to sex and occurrence of death, reported in individual case safety reports (ICSRs) in the years 2020–2021–2022–2023. Data are reported as the percentage of the total number of ICSRs (cases) (*n* = 209).

Sex reported in serious ICSRs with SARs to evolocumab	49.3%/50.7% (females/males)
Death reported in serious ICSRs as outcome of SARs to evolocumab	1/209

**Table 2:** Serious suspected adverse reactions (SARs) to evolocumab in European Economic Area (EEA) and United Kingdom signaled to EudraVigilance in the years 2020–2021–2022–2023. Total number of serious SARs = 258.

Adverse Reaction	Number of SARs	% of Total Number of Serious SARs
Myalgia	23	8.9
Flu-like symptoms	12	4.6
Diarrhea	9	3.5
Joint pain	7	2.7
Back pain	7	2.7
Urticaria	7	2.7
Angioedema	7	2.7
Drug effect incomplete/Lack of drug effect	7	2.7
Myocardial infarction/ischemia	6	2.3
Blood glucose increased	6	2.3
Breathlessness/Dyspnoea	6	2.3
Pruritus + Itchy + itchy rash + itching all over	6	2.3
Injection site reactions	6	2.3
Neuropathy	6	2.3
Acute pancreatitis	5	1.9
Herpes infection	4	1.5
Thrombocytopenia/Thrombocytopenia aggravated	4	1.5
Weight increase	4	1.5
Vomiting	4	1.5
Coronary artery disease aggravated/Coronary artery disease progression	4	1.5
Exanthema	4	1.5
Uveitis	4	1.5
Aortic stenosis	3	1.2
Rhabdomyolisis	3	1.2
Edema of legs + Edema	3	1.2
Deep-vein thrombosis	3	1.2
Pulmonary embolism	3	1.2
Hypoglycemia	3	1.2
Liver function test increased	3	1.2
Sleeplessness	3	1.2

Only serious suspected adverse reactions signaled more than two times are included in the table.

**Table 3:** Signals of suspected adverse reactions (SARs) to evolocumab by reaction group signaled to EudraVigilance from European Economic Area (EEA) and United Kingdom in the years 2020–2021–2022–2023, according to System Organ Class (SOC) level and sex distribution. Total number of serious SARs = 258.

Reaction Groups According to System Organ Class (SOC) Level	Males	Females	Males in All the SARs by the Reaction Group	Females in All the SARs by the Reaction Group	Total Number of SARs
Musculoskeletal and connective tissue disorders	48.9%	51.1%	8.9%	9.3%	47
General disorders and administration site conditions	38.7%	61.3%	4.6%	7.4%	31
Skin and subcutaneous tissue disorders	42.3%	57.7%	4.3%	5.8%	26
Gastrointestinal disorders	47.8%	52.2%	4.3%	4.6%	23
Vascular disorders	45.0%	55.0%	3.5%	4.3%	20
Cardiac disorders	41.2%	58.8%	2.7%	3.9%	17
Investigations	56.2%	43.7%	3.5%	2.7%	16
Metabolism and nutrition disorders	25.0%	75.0%	1.5%	4.6%	16
Nervous system disorders	23.1%	76.9%	1.2%	3.9%	13
Respiratory, thoracic, and mediastinal disorders	37.5%	62.5%	1.2%	1.9%	8
Psychiatric disorders	62.5%	37.5%	1.9%	1.2%	8
Eye disorders	28.6%	71.4%	0.77%	1.9%	7
Immune system disorders	50.0%	50.0%	1.2%	1.2%	6
Infections and infestations	40.0%	60.0%	0.77%	1.2%	5
Ear and labyrinth disorders	20.0%	80.0%	0.39%	1.5%	5
Blood and lymphatic system disorders	20.0%	80.0%	1.5%	0.39%	5
Renal and urinary disorders	60.0%	40.0%	1.2%	0.77%	5
SARs (Total)			112 (43.4%)	146 (56.6%)	258

**Table 4:** Evaluation of odds ratio for Individual Cases Safety Reports (ICSRs) with more than one suspected adverse reaction (SARs) to evolocumab in the European Economic Area (EEA) and the United Kingdom displayed in EudraVigilance for the years 2020–2021–2022–2023. Total number of ICSRs = 209.

	Males (*n* = 103)	Females (*n* = 106)	OR 95% CI	*p* Value
Number of cases with more than one adverse reaction	43/103	61/106	0.53 (0.31–0.92)	*p* = 0.023

Data are expressed as number of ICSRs related to evolocumab reporting one or more suspected adverse reactions. *p* was considered significant when <0.05.

**Table 5:** Serious suspected adverse reactions (SARs) associated with evolocumab use in European Economic Area (EEA) and United Kingdom displayed in EudraVigilance for the years 2020–2021–2022–2023 according to System Organ Class (SOC) level and age distribution of groups 18–64 years (adults) and 65–85 years (elders). Total number of SARs = 258; total number of SARs in the age groups of 18–64 and 65–85 years = 245.

SOC	18–64 Years (*n* = 113)	65–85 Years (*n* = 141)	*p* Value
Musculoskeletal and connective tissue disorders	20	27	N.S.
General disorders and administration site conditions	12	19	N.S.
Skin and subcutaneous tissue disorders	10	16	N.S.
Gastrointestinal disorders	7	16	N.S.
Vascular disorders	10	8	N.S.
Cardiac disorders	12 *	5	0.01249
Investigations	7	8	N.S.
Metabolism and nutrition disorders	5	10	N.S.
Nervous system disorders	5	8	N.S.
Respiratory, thoracic, and mediastinal disorders	5	3	N.S.
Psychiatric disorders	1	7	N.S.
Eye disorders	5	2	N.S.
Immune system disorders	3	3	N.S.
Infections and infestations	2	3	N.S.
Ear and labyrinth disorders	4	1	N.S.
Blood and lymphatic system disorders	2	3	N.S.
Renal and urinary disorders	3	2	N.S.

Only serious suspected adverse reactions of people aged 18–41 years and 65–85 years are included in the table. * *p* was considered significant when <0.05.

**Table 6:** Reporting odds ratio (ROR) values of serious suspected adverse reactions (SARs) related to the prescription of Proprotein Convertase Subtilisin/Kexin type 9 (PCSK9) inhibitors evolocumab and alirocumab in the European Economic Area and the United Kingdom in the years 2020–2021–2022–2023.

SOC	SARS to Evolocumab *n* = 258	SARs to Alirocumab *n* = 259	ROR (C.I.)
Musculoskeletal and connective tissue disorders	24	29	0.79 (0.44–1.39)
General disorders and administration site conditions	31	40	0.72 (0.43–1.19)
Skin and subcutaneous tissue disorders	26	34	0.72 (0.42–1.23)
Gastrointestinal disorders	23	27	0.81 (0.45–1.46)
Vascular disorders	20	13	1.54 (0.75–3.16)
Cardiac disorders	17	14	1.19 (0.58–2.48)
Investigations	16	20	0.76 (0.39–1.51)
Metabolism and nutrition disorders	16	8	2.00 (0.84–4.76)
Nervous system disorders	13	21	0.58 (0.28–1.19)
Respiratory, thoracic, and mediastinal disorders	8	15	0.50 (0.21–1.21)
Psychiatric disorders	8	5	1.57 (0.51–4.88)
Eye disorders	7	5	1.37 (0.43–4.38)
Immune system disorders	6	9	0.64 (0.22–1.83)
Infections and infestations	5	7	0.69 (0.22–2.20)
Ear and labyrinth disorders	5	2	2.46 (0.47–12.8)
Blood and lymphatic system disorders	5	4	1.22 (0.32–4.60)
Renal and urinary disorders	5	6	0.81 (0.24–2.68)

C.I.: confidence intervals.

## Data Availability

The data presented in this study are contained within the article. Members of the public can access the EudraVigilance database for adverse reactions.
